# Berlin Heart EXCOR as a Bridge to Transplantation in Pediatric End-Stage Heart Failure: A Retrospective Cohort Study

**DOI:** 10.3390/jcdd12120465

**Published:** 2025-11-29

**Authors:** Mohannad Dawary, Dimpna Brotons, Felix W. Tsai

**Affiliations:** 1 Cardiac Surgery, Heart Center, King Faisal Specialist Hospital & Research Center, Riyadh 13338, Saudi Arabia; mohanad.aldawary@gmail.com; 2 Pediatric Cardiology, Heart Center, King Faisal Specialist Hospital & Research Center, Riyadh 11211, Saudi Arabia; dimpnacalila@gmail.com

**Keywords:** heart transplant, Berlin Heart, heart failure, pediatric

## Abstract

Background: Ventricular assist devices serve as a critical bridge to transplantation for pediatric patients with end-stage heart failure. This study evaluated the outcomes of pediatric patients who received Berlin Heart EXCOR support for end-stage heart failure. Methods: We retrospectively analyzed data from 11 consecutive pediatric patients (63.64% male, median age 60 months) who underwent Berlin Heart implantation from November 2021 to April 2025. The majority (90.90%) had dilated cardiomyopathy, and 72.73% were INTERMACS class I. Results: Of the 11 patients, 54.54% received an LVAD only, 36.36% received a BiVAD, and 9.09% required an LVAD followed by an RVAD. The postoperative mean ICU stay was 140 ± 73 days, and total hospital stay was 192 ± 96 days. Significant post-implant complications included stroke (27.27%), bleeding requiring exploration (27.27%), and pneumonia (36.36%). Ten patients (90.91%) were successfully bridged to heart transplantation, with one pre-transplant mortality (9.09%) due to brain hemorrhage. The median time to transplantation was 88 days (interquartile range, IQR: 78–177). During a median follow-up of 17 months (IQR: 7–32), two patients died post-transplant, resulting in an overall survival rate of 67.50% at 3 years. Conclusions: Despite significant complications and prolonged hospitalization, the Berlin Heart demonstrated effectiveness as a mechanical circulatory support device for pediatric patients, with a high rate of successful bridging to transplantation and acceptable mid-term survival. These findings support its use as a viable bridge to transplantation in pediatric end-stage heart failure.

## 1. Introduction

Heart failure in children is a leading cause of morbidity and mortality [1]. The pediatric population with heart failure differs substantially from adults, with congenital heart defects being a predominant cause in this subset of patients [2,3]. A previous study demonstrated that congenital heart disease was the cause of pediatric heart failure in more than half of the cases [4]. Although heart transplantation is the gold standard treatment for end-stage heart failure in children, it has significant limitations. These constraints are particularly pronounced in the pediatric population, where suitable donor organs are scarce [5,6] and mortality is high on the transplant waiting list [7]. Consequently, mechanical circulatory support devices have emerged as critical alternatives for managing advanced heart failure in children [8]. The Berlin Heart EXCOR (Berlin Heart GmbH, Berlin, Germany) is a pulsatile, pneumatically driven extracorporeal system that offers various pump sizes, making it uniquely adaptable to the pediatric population with end-stage heart failure as a bridge to transplantation [7,9]. Despite its widespread use, comprehensive data regarding the outcomes and complications associated with Berlin Heart implantation in pediatric patients remain limited. Previous studies have reported variable results, with an average success rate for bridging to transplantation of 77% and significant complication rates, particularly for neurological events and bleeding [10,11]. The heterogeneity of the pediatric population, variations in institutional protocols, and the relatively small number of reported cases contribute to the challenges in establishing definitive outcome measures. Understanding the efficacy and safety profile of the Berlin Heart in pediatric patients is crucial for optimizing patient selection, perioperative management, and long-term care strategies. This knowledge gap highlights the need for ongoing research on the use of this device in children with end-stage heart failure. The present study aimed to evaluate the outcomes and complications associated with Berlin Heart implantation in pediatric patients as a bridge to transplantation at a single tertiary referral cardiac center.

## 2. Materials and Methods

### 2.1. Design and Patients

This retrospective cohort study involved 11 consecutive pediatric patients who underwent Berlin Heart EXCOR implantation for end-stage heart failure at a single tertiary cardiac center from November 2021 to April 2025. All patients who received Berlin Heart support during this period were included in the analysis. The study focused exclusively on pediatric patients (age < 14 years) who required mechanical circulatory support as a bridge to transplantation.

### 2.2. Data Collection

Comprehensive data were collected from electronic medical records, including demographic information, clinical characteristics, operative details, postoperative course, complications, and follow-up outcomes. Baseline data included age, sex, body surface area (BSA), primary diagnosis, comorbidities, INTERMACS (Interagency Registry for Mechanically Assisted Circulatory Support) profile [12], preoperative right ventricular function, left ventricular internal diameter at diastole (LVIDd), and preoperative extracorporeal membrane oxygenation (ECMO) use. Operative data encompassed device configuration (LVAD, RVAD, or BiVAD), cardiopulmonary bypass time, and aortic cross-clamp time when applicable. Postoperative outcomes included length of stay in the intensive care unit (ICU) and hospital, postoperative right ventricular function, anticoagulation regimen, and complications such as stroke, bleeding, infection, renal failure requiring dialysis, and device-related issues. Long-term outcomes focused on successful bridging to transplantation, time to transplantation, post-transplant survival, and overall survival. Follow-up data were collected through the last clinical encounter or death.

### 2.3. Ethical Considerations

This study was conducted in accordance with the ethical principles outlined in the Declaration of Helsinki [13] and the institutional policies governing retrospective research. The study protocol was reviewed and approved by the institutional review board, and the requirement for individual patient or guardian consent was waived due to the retrospective nature of the analysis and the use of de-identified data.

### 2.4. Statistical Analysis

Continuous variables were expressed as mean ± standard deviation when normally distributed or as median with interquartile range (25–75th percentiles) when non-normally distributed. Categorical variables were presented as frequencies and percentages. The Shapiro–Wilk test was used to assess the normality of continuous data distribution. Survival analysis was performed using the Kaplan–Meier method, with time calculated from Berlin Heart implantation to death or last follow-up. For overall survival, the endpoint was death from any cause. All statistical analyses were performed using Stata version 18 (Stata Corp., College Station, TX, USA).

## 3. Results

### 3.1. Baseline Data

The study included 11 pediatric patients, with a male predominance (63.64%). The median age was 5 years. The mean BSA was 0.68 ± 0.25 m^2^. The majority of patients (90.90%) had dilated cardiomyopathy as their primary diagnosis, with one patient (9.09%) having systolic heart failure. Several patients had notable comorbidities, including very long-chain acyl-CoA dehydrogenase deficiency (18.18%), Kawasaki disease (9.09%), and Shone’s complex (9.09%). Most patients (72.73%) were classified as INTERMACS profile I (critical cardiogenic shock), while 27.27% were profile 2 (progressive decline despite inotropic support). The mean left ventricular internal diameter at diastole was 4.81 ± 1.05 cm. The demographic and clinical characteristics of these patients are summarized in Table A1, Appendix A.

### 3.2. Operative and Postoperative Outcomes

All patients underwent Berlin Heart implantation via median sternotomy using cardiopulmonary bypass (CPB) with mild hypothermia (34 °C). The mean cardiopulmonary bypass time was 225 ± 58 min. Aortic cross-clamping was performed in five patients with small ascending aortas with a median cross-clamp time of 33 min.

Regarding device configuration, the majority of patients (54.54%) received left ventricular assist device (LVAD) support only, while 36.36% required biventricular support (BiVAD) from the outset. One patient (9.09%) initially received LVAD support but subsequently required right ventricular assist device (RVAD) implantation. This was performed via redo sternotomy without the need for CPB.

All patients remained in the hospital before undergoing orthotopic heart transplant. The postoperative course had a mean ICU stay of 140 ± 73 days and total hospital stay of 192 ± 96 days. Neurological complications were notable, with strokes occurring in 27.27% of patients. Bleeding requiring surgical exploration occurred in 27.27% of cases, and bleeding episodes of any kind were observed in 54.54% of patients, including subdural hematoma (4 patients), oral bleeding (1 patient), and hematuria with abdominal omental hemorrhage (1 patient). Importantly, no gastrointestinal bleeding or device thrombosis was reported. Infectious complications included pneumonia in 36.36% of patients and driveline infections in 18.18%. One patient (9.09%) required dialysis for renal support. Liver function tests showed median ALT of 34 U/L (IQR: 14–105) and AST of 51 U/L (IQR: 26–118). Postoperative right ventricular function improved compared to preoperative status, with 63.64% of patients having normal function and 36.36% having only mildly reduced function. 

All patients received anticoagulation therapy, with various regimens including heparin alone (36.36%), heparin and warfarin (27.27%), argatroban and warfarin (27.27%), or argatroban alone (9.09%), based on patient response. Bivalrudin is not available at our institution.

Summation of operative details and outcomes are summarized in Table A2, Appendix A.

### 3.3. Long-Term Follow-Up

**Figure 1:**
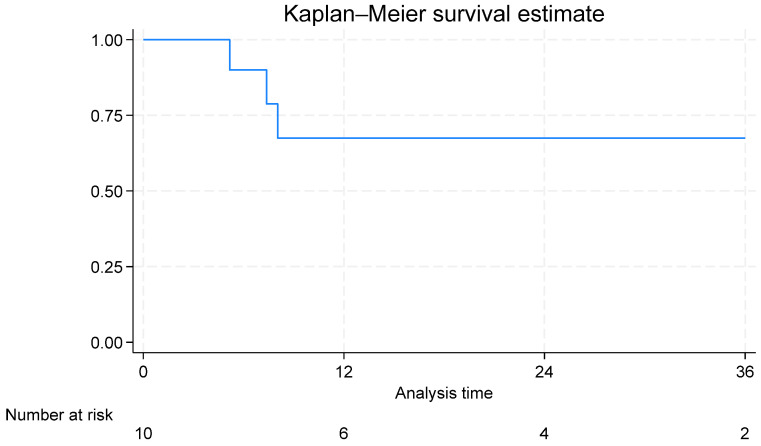
Kaplan–Meier curve for overall survival after Berlin Heart EXCOR.

Of the 11 patients who received Berlin Heart support, 10 patients (90.91%) were successfully bridged to heart transplantation. One patient (9.09%) died before transplantation due to brain hemorrhage, which represents the only pre-transplant mortality in our cohort. The median time to transplantation was 88 days (25–75th percentiles: 78–177 days). The median follow-up period was 17 months (25–75th percentiles: 7–32 months). During this follow-up period, 2 patients died after transplantation. The remaining 8 patients (80% of those transplanted) were alive at the last follow-up. Survival at 3-year post-Berlin Heart was 67.50% (Figure 1).

## 4. Discussion

This retrospective cohort study evaluated the outcomes and complications of Berlin Heart ventricular assist device implantation in 11 consecutive pediatric patients with end-stage heart failure. The cohort had a median age of 5 years, with a male predominance. The majority of patients (90.90%) had dilated cardiomyopathy as their primary diagnosis, and most (72.73%) were classified as INTERMACS profile I, indicating critical cardiogenic shock. The postoperative course was characterized by prolonged hospitalization and significant complications, including stroke (27.27%), bleeding requiring exploration (27.27%), and pneumonia (36.36%). Despite these challenges, 10 patients (90.91%) were successfully bridged to heart transplantation, with only one pre-transplant mortality. The overall survival rate at 3 years post-Berlin Heart implantation was 67.50%.

The Berlin Heart EXCOR has been a cornerstone of mechanical circulatory support for pediatric patients since its FDA approval in 2011 [14,15,16]. Our findings regarding the high rate of successful bridging to transplantation (90.9%) compare favorably with those of previous studies. Almond et al. reported outcomes of Berlin Heart EXCOR in 204 US children, which found a successful bridging or recovery rate of 75% [10]. Similarly, Fraser et al. demonstrated higher survival rates in patients with a Berlin Heart EXCOR device bridged to transplant compared to those bridged on ECMO [17]. Zafar et al. reported a success rate with Berlin Heart (Defined as transplanted, recovery, or 180-day survival) of 86% and 76% [9].

The neurological complication rate in our cohort (27.27%) aligns with previous reports. The Berlin Heart investigational device exemption trial reported neurological events in 26.5% of the patients [18]. Jordan et al. studied 204 patients from 47 centers and reported that 29% of patients had at least one neurological event with Berlin Heart EXCOR [19]. Similarly, Zafar et al. reported 32% and 24% neurological complications with Berlin Heart BiVAD and LVAD, respectively [20]. These findings underscore the persistent challenge of neurological complications in pediatric patients supported with ventricular assist devices. The high incidence of neurological events may be attributed to several factors, including the challenges of maintaining optimal anticoagulation in children, the pulsatile nature of the Berlin Heart device, and the critical pre-implantation status of many patients that could have affected the brain before implantation. 

Given the significant anticoagulation required, bleeding complications were also significant in our cohort, with 27.27% requiring surgical exploration and 54.54% experiencing bleeding episodes of any kind. These rates are consistent with those found in previous studies. Huang et al. reported episodes of major bleeding in 39% of patients on Berlin Heart EXCOR [21]. The bleeding risk is particularly pronounced in the early post-implantation period and is often related to the intensive anticoagulation regimens required to prevent thromboembolic complications [22]. However, Rosenthal and associates reported that stroke was lower after triple antithrombotic therapy following Berlin Heart compared to dual therapy, with no increased risk of bleeding [23]. Rohde et al. reported that lower BSA was a risk factor for bleeding following Berlin Heart implantation [24]. Warfarin was the main anticoagulant used in our series.

Infectious complications, particularly pneumonia (36.36%) and driveline infections (18.18%), represent another significant challenge in the management of these patients. Infection complications are common in pediatric patients with Berlin Heart support. Munoz et al. reported 36 infectious episodes in 13 out of 15 patients who underwent Berlin Heart EXCOR implantation [25]. Auerbach et al. reported infectious complications in 17% of patients after Berlin Heart implantation, with the most common among these infections being device-related (51%) [26]. Prolonged hospitalization and device-related factors contribute to this elevated risk of infection.

Despite these complications, our study demonstrated a remarkably high rate of successful bridging to transplantation (90.91%), which exceeds many previously reported rates [9]. This success may be attributed to several factors, including careful patient selection, meticulous perioperative management, and a dedicated multidisciplinary team approach. The median time to transplantation in our cohort (88 days) is comparable to that reported in other studies, reaching up to 800 days [20,22,27]. The overall survival rate at 3 years post-Berlin Heart implantation (67.50%) in our cohort is encouraging and consistent with previous reports. Almond et al. reported 75% survival rate after one year of Berlin Heart implantation [10]. These findings suggest that despite the significant complications associated with Berlin Heart support, the long-term outcomes for patients who successfully undergo transplantation are generally favorable.

The findings of this study have several important clinical implications. First, they demonstrate the effectiveness of the Berlin Heart as a bridge to transplantation in pediatric patients with end-stage heart failure, achieving a high success rate, even in critically ill patients (INTERMACS profile I). This supports the continued use of this device in the pediatric population, particularly when donor availability is limited. Second, the significant complication rates, especially neurological events and bleeding, highlight the need for vigilant monitoring and management strategies. Optimizing anticoagulation protocols, implementing standardized neurological surveillance, and developing strategies to minimize the risk of bleeding are critical areas for improvement. Third, the extended hospitalization seen in our cohort highlights the resource-intensive nature of managing these patients. This has implications for healthcare resource allocation, family support systems, and quality of life considerations. Prioritizing the development of strategies to reduce hospital length of stay while ensuring safety and effectiveness should be a focus for future research. Finally, the favorable post-transplant survival rates suggest that despite the challenges and complications associated with Berlin Heart support, the long-term outcomes for patients who successfully undergo transplantation are generally good. This provides valuable prognostic information for clinicians counseling families about the expected outcomes of this therapeutic approach.

## 5. Limitations

This study has several limitations that should be acknowledged. First, the small sample size (n = 11) limits the statistical power and generalizability of our findings. Second, the single-center design may not reflect practices or outcomes at other institutions. Third, the retrospective nature of the study introduces potential biases in data collection and analysis. Fourth, the relatively short follow-up period (median 17 months) may not capture long-term complications or survival trends. Finally, the absence of a control group precludes direct comparisons with alternative management strategies. Despite these limitations, our study provides valuable insights into the real-world outcomes of Berlin Heart support in pediatric patients and contributes to the growing body of evidence regarding the effectiveness and safety of this therapeutic approach.

## 6. Conclusions

The Berlin Heart ventricular assist device demonstrates effectiveness as a mechanical circulatory support option for pediatric patients with end-stage heart failure, with a high rate of successful bridging to transplantation. Despite significant complications and prolonged hospitalization, our study revealed a high success rate in bridging to heart transplantation. Neurological events, bleeding complications, and infections continue to be significant challenges in the management of these patients, underscoring the need for vigilant monitoring and optimized management protocols.

Despite these challenges, the Berlin Heart provides a viable bridge to transplantation for pediatric patients with end-stage heart failure, offering hope for this vulnerable population when donor availability is limited. Future research should focus on strategies to minimize complications, optimize anticoagulation protocols, and improve the quality of life for these patients during the bridging period. Multicenter, prospective studies with larger sample sizes are needed to further validate these findings and establish standardized management protocols for pediatric patients requiring mechanical circulatory support.

## Data Availability

The Authors confirm that the data supporting the findings of this study are available within the article.

## Abbreviations

The following abbreviations are used in this manuscript:

INTERMACS	Interagency Registry for Mechanically Assisted Circulatory Support
LVIDd	left ventricular internal diameter at diastole
ECMO	extracorporeal membrane oxygenation
ICU	intensive care unit
LVAD	left ventricular assist device
BiVAD	Biventricular support
RVAD	Right ventricular assist device
BSA	Body surface area
CPB	Cardiopulmonary bypass

## Appendix A

**Table A1:** Baseline data of pediatric patients who received Berlin Heart EXCOR.

Variables	(n = 11)
Male	7 (63.64%)
Age, months	60 (22–96)
BSA, m^2^	0.68 ± 0.25
**Comorbidities**	
Very long-chain acyl-CoA dehydrogenase deficiency	2 (18.18%)
Kawasaki	1 (9.09%)
Shone’s complex	1 (9.09%)
**Blood group**	
A+	2 (18.18%)
B+	2 (18.18%)
O+	6 (54.55%)
O−	1 (9.09%)
**Right ventricular dysfunction**	
Normal	3 (27.27%)
Mild dysfunction	2 (18.18%)
Moderate dysfunction	4 (36.36%)
Severe dysfunction	2 (18.18%)
Previous cardiac surgery (arch and coarctation repair)	1 (9.99%)
Preoperative anticoagulation	8 (72.73%)
	7 heparin
	1 Argatroban
Pre ECMO	9 (81.82%)
**Diagnosis**	
Dilated cardiomyopathy	10 (90.90%)
Systolic heart failure	1 (9.09%)
**INTERMACS class**	
I	8 (72.73%)
II	3 (27.27%)
LVIDd (cm)	4.81 ± 1.05

BSA: body surface area; ECMO: extracorporeal membrane oxygenation; LVIDd: left ventricular internal diameter at diastole. Data are presented as mean (SD), median (IQR) or numbers (%).

**Table A2:** Operative and postoperative data of pediatric patients who received Berlin Heart EXCOR.

Variables	(n = 11)
CPB time, min	225 ± 58
Cross-clamp time, min (n = 5)	33 (29–33)
**Device configuration**	
LVAD	6 (54.5450
BiVAD	4 (36.36%)
LVAD then RVAD	1 (9.09%)
ICU stay, d	140 ± 73
Hospital stay, d	192 ± 96
Stroke	3 (27.27%)
Bleeding requiring exploration	3 (27.27%)
Dialysis	1 (9.09%)
ALT, U/L	34 (14–105)
AST, U/L	51 (26–118)
Pneumonia	4 (36.36%)
Driveline infection	2 (18.18%)
Any bleeding episodes	6 (54.54%)
**Postoperative right ventricular function**	
Normal	7 (63/64%)
Mildly reduced	4 (36.36%)
**Postoperative anticoagulation**	
Heparin	4 (36.36%)
Heparin and warfarin	3 (27.27%)
Argotroban+ warfarin	3 (27.27%)
Aegatroban	1 (9.09%)

CPB: cardiopulmonary bypass; LVAD: left ventricular assist device; RVAD: right ventricular assist device; BiVAD: biventricular assist device. Data are presented as mean (SD), median (IQR), or numbers (%).
